# From bubbling to circulating fluidized bed combustion—development and comparison

**DOI:** 10.1016/j.heliyon.2024.e33415

**Published:** 2024-06-21

**Authors:** Bo Leckner

**Affiliations:** Division of Energy Technology, Chalmers University of Technology, 41296, Göteborg, Sweden

**Keywords:** Fluidized bed conversion, Bubbling fluidized bed, Circulating fluidized bed, Sulphur capture, NOx emission

## Abstract

The most significant introduction of fluidized bed combustion technology took place about 50 years ago. Initially the combustion beds were of the bubbling type. Once the designs had reached commercial application, several drawbacks were discovered: Erosion on in-bed heat-exchanger tubes, insufficient combustion and desulphurization efficiencies with coal, unfavourable scale-up to electric utility-size. The problems were solved by applying circulating systems. The present text compares these bubbling and circulating designs. It is concluded that the bubbling bed may not be suitable for coal combustion, but for biomass and organic waste most of the drawbacks disappear, and the bubbling bed, being simpler, may have an economic advantage over CFB that should be considered. In addition, combinations of CFB and BFB are quite favourable in many applications.

## Highlights

• Bubbling fluidized bed has been shown insufficient for the combustion of coal.
• Circulating fluidized bed is dominant for combustion of all kinds of solid fuel.
• Bubbling beds may have an advantage for the combustion of biomass and waste.
• Combinations of bubbling and circulating beds are quite favourable.

## 1 Introduction

Fluidized bed (FB) was first applied for gasification, starting 100 years ago. Below, gasification will be mentioned, but the problems to be treated are mostly occurring in boilers. Therefore, the emphasis is on combustion, where the development was strongly enhanced about 50 years ago.

It is easy to understand why FB became of interest for the combustion of solid fuels, first sporadically, already in the 1950s, and then more intensely in the 1970s, while it promised to alleviate the then obvious environmental problems (particularly the flue-gas emissions of sulphur and nitrogen oxides) created by the increasing industrialization and the related energy demand [1]. FB is a fuel converter where a hot mass of inert fluidized particles serves as a stable environment for combustion. Moreover, its heat transfer capacity is outstanding, and the FB (then bubbling bed, BFB) arrangement appeared ideal for the aims to be fulfilled: efficient combustion of solid fuels, high rate of heat transfer from the bed to heat-transfer surfaces in the bed, low emission of sulphur dioxide when using limestone for sulphur capture, and low emission of nitrogen oxide (NO). Thermal NO, causing problems in pulverized fuel combustors, is avoided because of the low temperature maintained in an FB boiler (800–900 °C).

In the early development, combustion experiments were carried out in laboratory environments or theoretically, and everything appeared promising. However, when the first commercial boilers had been operated for some time and gained experience, several problems appeared: erosion on heat-transfer tubes in the bed, insufficient combustion efficiency, and difficulties in scale-up to sizes suitable for production of electric power [1]. The problems mentioned were remedied largely by a transition in the design that took place in the 1980s. In the new design, the circulating fluidized bed (CFB) boiler, the fluidization velocity was increased, and that enhanced the combustion in terms of surface power (MW/m^2^ bed surface-area), facilitating scale-up. Furthermore, the heat-transfer surfaces in the CFB boiler were aligned in the vertical direction, parallel to the main flow of particles in the upper part of a tall furnace, and this greatly reduced the danger for erosion. The surfaces obtained their heat from the entrained bed particles, recirculated through a cyclone and returned to the bottom of the furnace, thus reducing the loss of unburned char. This CFB boiler subsequently became the dominant design.

This transition came 40 years ago. Consequently, the young generation of engineers and scientists in the field are mostly aware of the CFB boiler, and the BFB boiler is forgotten or appears less important. Sometimes, a CFB boiler is proposed for operation under circumstances where a BFB boiler could have been an equivalent and cheaper alternative. Therefore, it is the purpose of this overview to investigate the differences and similarities between the two kinds of boiler, based on available experience, and to identify conditions of operation, under which a BFB may be advantageous, as well as those where BFB should not be used. Moreover, a comparison between BFB and CFB can lead to a better understanding of the presently more important CFB design. The comparison is focused on the items mentioned, and the intention has not been to make a comprehensive review on the development of BFB and CFB boilers. Moreover, much of the background was obtained at Chalmers University of Technology, Sweden, because of the unique opportunity for the present comparison of having access to rather large research installations, a 16 MW_th_ BFB boiler and a 12 MW_th_ CFB boiler.

## 2 Definitions and regimes of operation

A bubbling combustion bed of Group B particles is maintained when the superficial velocity is above the minimum one, u_mf_. The fluidization velocity is both fluidizing the bed and delivering oxygen for combustion, so it is desired to have a high velocity to obtain a high output per unit cross-section area of the bed. Because of interaction between particles and bypass through bubbles it is uncertain to determine the maximum velocity for which a bubbling bed can exist. What happens with the fluidization when the velocity increases? One well defined velocity limit is the terminal velocity, the limit above which a single spherical particle can be carried away by the gas. However, there is no doubt that a bubbling bed can exist at higher velocities than that. According to experience documented in literature, at increasing velocity there is a transition in the state of fluidization from bubbling to turbulent fluidization and then, during further rise of velocity, to fast fluidization of an entrained bed. Bi et al. [2] mention three types of bubbling-turbulent transition, the first one occurs because of bubble splitting in a bed of Group A particles, the second one because of slugging in Group B beds. With further increase in velocity, turbulent fluidization is observed. The third type of transition is not of interest here. None of these transition types are likely to occur in a combustion bed, consisting of Group B particles and being much wider than the possible bubble size. Slugging fluidization cannot exist under such conditions. Bubbles in beds of Group B particles increase in size with velocity and may become large, but they are eventually limited by the height of the dense bed (and not by the walls of the fluidization vessel, like in narrow experimental facilities). The bed and the bubbles can stand an increasing velocity because of through-flow of gas through the bubbles [3]; most particles in the particle phase of the bed are not exposed to extreme velocities as a result of the escape of gas in the by-pass flow through the bubbles, but despite this, elutriation of particles from the bed does increase with velocity.

The elutriation is a limiting feature of a bubbling bed. When the carry-over of particles becomes severe the vessel will be emptied, the bed disappears. Because of fragmented char particles and left-over ashes in the bed, the particle size distribution in a combustion bed will always be wide and contain a considerable fraction of small particles that can be carried away by the gas. The particles are carried away depending on their size. If the velocity is high, even the larger particles tend to disappear from the dense bed. There are only two solutions to avoid excessive carry-over; using a lower velocity or arranging for particle recirculation. At high velocity and small particles, if the particles are separated from the gas and recirculated, irrespective of the dominant fluidization behaviour of the bed, the system becomes a circulating bed, a CFB. So, a bubbling bed could be a circulating bed if some particles are elutriated and there is a particle separator to return the particles to the bed. This description differs from the general understanding of BFB or CFB, as is illustrated by one of the published definitions of CFB, “A bed is bubbling or circulating, depending on whether the bed material remains in place or is transported out of the vessel by the fluidizing gas, and then recovered and returned to the bed.” [4]. In contrast to the definitions, it seems that a bubbling bed also can be a circulating bed, or vice versa, a circulating bed also can be bubbling. So, one has to destinguish between type of device (BFB or CFB) and fluidization regimes of the media in these devices.

There are few measurements on boilers that can verify the above remarks on the state of fluidization. However, pressure fluctuations were measured on the 12 MW_th_ CFB boiler at Chalmers University [3] at velocities up to above 5 m/s without attaining a transition from a bubbling to a turbulent bed as described by published definitions on the properties of this transition. Sekret and Nowak [5] measured the bottom-bed height and pressure fluctuations for velocities up to 8 m/s in a 670 MW CFB boiler burning coal. They classified their observed bed as “bubbling-turbulent”, probably because they found a maximum on the measured pressure fluctuation-velocity curve. Fortunately, they also measured the variation of the bottom-bed height very carefully. Since this parameter decreased with velocity (as is reasonable) at the same time as the pressure fluctuation, the most likely interpretation is that the pressure fluctuation decreased because of smaller bubbles at a lower bed height during increasing velocity, and then the maximum observed on the pressure fluctuation – velocity curve was an artefact caused by the simultaneous reduction of the bottom-bed height. Hence, the bed was not turbulent but still bubbling despite the high gas velocities.

Conclusion: it is the recirculation rather than the mode of fluidization that distinguishes BFB from CFB.

## 3 .The design of BFB and CFB boilers

**Fig. 1:**
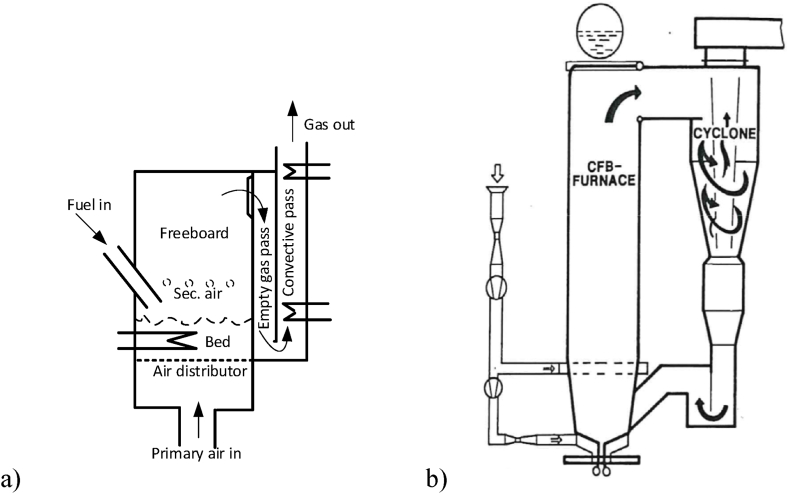
a) A BFB boiler. An empty gas pass fulfils the European regulation for waste combustion, requiring a gas-residence time of 2 s downstream of the last supply of air, b) A CFB boiler. The air-supply system is seen. The fuel feed arrangement is a screw feeder, and the back pass (not shown), downstream of the particle separator, is like that of the BFB boiler. In both boilers the walls are covered by membrane tubes. There are internal heat-transfer tubes in the bed of the BFB but not in the CFB boiler.

Typical features of BFB and CFB boilers are shown in Fig. 1. The operation data of the boilers are summarized in Table 1. Both boilers on the figure are representative in general but small; the BFB was built as a demonstration unit at Chalmers University of Technology, a steam boiler burning coal, having a thermal power of 16 MW_th_ [6]. The other was also a steam boiler operated with coal, erected in a district-heating system in Sweden with a thermal power of 40 MW_th_ at maximum continuous load. The boilers are chosen for comparison because they have the same bed-surface area, 10 m^2^. This means that they have the surface powers of 1.6 MW_th_/m^2^ and 4 MW_th_/m^2^ cross-sectional area, respectively, which reveals one of the main problems with BFB compared to CFB; the surface power of BFB is relatively low, and the size of the bed becomes prohibitively large at up-scaling to larger sizes. As a result, BFB is limited to the size of a few hundreds MW_th_, while CFB could reach thousands MW_th_ (the biggest now has an electric power of about 660 MW_e_, which translates to a thermal power of about between 1500 and 1700 MW_th_).

**Table 1:** The most important data for the BFB and the CFB boilers to be compared.

Parameter	BFB	CFB
Size, MW_th_	16	40
Bed temperature, ^o^C	850	850
Fluidization velocity, m/s	2	6
Bed cross-section surface, m^2^	10	10
Bed material size, mm		0.1–0.3
Bed height (m), pressure drop (kPa)	1, 10	30^b^, 10
Excess-air ratio, %	30	20
Primary:secondary air ratio, example^a^,-	60:40	60:40
Fuel	Coal	Coal

a See comment in text.

b Expanded bed, including bottom bed, splash zone, and transport zone.

Table 1 shows that, typically, the bed of a BFB boiler uses coarser particles than the CFB. In the BFB the carry-over of bed material (and char) is a problem that the operators and designers try to mitigate as much as possible. One way is by the choice of a coarser bed material, thereby reducing the elutriation.

### 3.1 Secondary air

Both boilers use secondary air. The ratios of air supply given in Table 1 are indicative only. The ratios can be adjusted according to the conditions of operation of the boilers. In later developments, BFB operated with coal may have higher primary air ratios, for example, 60–70 for lean coal and 80 % for anthracite, whereas the ratio may be lower than the value given in the table for biomass and high-volatile fuels, operating without heat transfer surface in the bed (see below).

Secondary air is used in most combustors. The primary reason is to create an oxygen-deficient zone that promotes the reduction of nitrogen oxides NO. In FBC there are additional reasons: to reduce the pressure drop by adding some air above the bottom bed, and to promote the combustion by increasing the mixing in the freeboard (BFB) or to maintain combustion by mixing and oxygen supply at higher levels in the furnace (CFB). Also, the rate of secondary air acts on the oxidising-reducing conditions of the bed, for promoting oxidation during sulphur capture from coal versus displacing combustion to the freeboard in the biomass case.

### 3.2 Heat transfer

In the coal-fired BFB the bed temperature is controlled by heat-transfer surfaces in the bed (see Fig. 1, Fig. 5b). In addition, the gas temperature of the freeboard is balanced by heat transfer to the surrounding membrane-tube walls. As in all boilers, the remaining heat is removed from the gases by convective heat exchangers in the back pass to reach a low temperature (about 150 °C) before entering the dust-cleaning devices and finally leaving through the stack. The first important operational experience of the BFB was received after a few thousand hours of operation: The in-bed heat-transfer surfaces were severely eroded and had to be removed. The boiler of Fig. 1a had to be downgraded to operation at a lower load, and finally it was stopped [7]. The results shown below were obtained before the downgrading.

In the CFB boiler such problems are avoided. The heat transfer surfaces can be located in the less dense regions of the furnace, aligned in parallel with the main flow of gas and particles in the form of wing-wall surfaces in addition to the membrane-tube walls of the furnace. Then, the danger for erosion is reduced, provided that excess velocities are avoided (5–6 m/s is the maximum according to experience). In addition, the most exposed parts of the furnace walls are covered by protective layers to avoid erosion damages. For geometrical reasons the available wall surface depends on the size of the furnace; a larger furnace has relatively seen a smaller wall-surface area. The size limit, above which the wall itself is not sufficient, is about 50 MW_th_, so the present CFB boiler, having a power of 40 MW_th_, only uses membrane-tube walls for heat transfer. Wing walls and other heat-transfer equipment in the furnace are needed only above that size. For the same reason, when the size of a CFB boiler exceeds about 300 MW_e_, also the space for internal surface extensions, such as wing walls, is not sufficient, and external heat-exchangers are required.

### 3.3 Combustion

The combustible part of solid fuel consists of fixed carbon and volatiles. When the fuel enters the hot fluidized bed, it first dries and then the volatiles are released, leaving char to be converted in the bed. The gaseous components are only partly reacted in the bed, but they mostly follow the fluidization gases into the freeboard where they burn, assisted by the secondary air. The char, remaining in the bed, is gradually consumed and ends up as a fragile char structure, which eventually breaks, and the fragments burn or are carried away by the fluidization gas. In the freeboard of a BFB, the impact of the combustible gases depends on the volatile content of the fuel. High-volatile fuels release a large amount of combustible gas whose burning could create hot zones in the freeboard. To avoid overheating and melting of ash particles the secondary air should be added with care, perhaps in steps organized as injection inlets in tiers at different heights. With low-volatile fuels, on the other hand, the elutriated ash and char particles meet a freeboard without much combustion and the temperature falls with height, cooled by radiation from the gas to the surrounding membrane-tube walls, [7,8]. In conclusion, overbed combustion of volatiles in a freeboard, only containing occasional particles elutriated from the bed, is a significant feature of BFB.

**Fig. 2:**
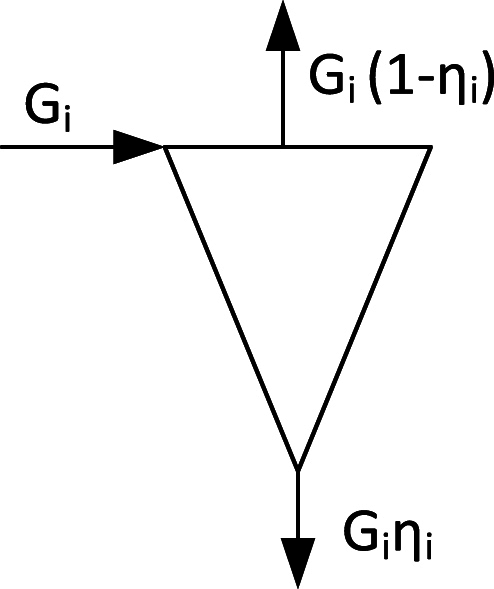
Particle separator with particle fluxes G_i_ of the size fraction i.

The same process as in the freeboard of a BFB occurs in the CFB, but it is not noted in the same way because of the considerable heat transport by the denser particle suspension in the splash and transport zones in the upper part of the riser, creating a suitable environment for combustion, also for fine char particles. (It was said above that the particle concentration in the upper part of the CFB furnace, the transport zone, is low. A typical particle volume concentration at full load could be only 0.007, but, still, this means 18 kg particles/m^3^gas. This is why it was called “considerable” with respect to heat transport), high enough to absorb and transport heat to and inside of the upper part of the furnace, avoiding hot spots and creating a reasonable environment for char (and volatiles) combustion. Also in the bottom bed, the combustion conditions are more favourable than those of a BFB because of a more expanded bed and an extended splash zone with improved mixing. Moreover, in the CFB unburned char particles are recirculated. Each turn of recirculation adds to the residence time of the particles in the hot furnace, hence promoting combustion and reducing the unburned char compared to the BFB. This can be illustrated from the simple sketch of a cyclone separator in Fig. 2. The circulating flux G_i_ of particles of size i enters the cyclone from the furnace. With the cyclone efficiency η_i_ for the size fraction i, the amount G_i_(1-η_i_) is lost in each particle-circulation turn, and G_i_η_i_ continues through a particle seal and returns to the furnace. Together, the input flux G_i_ will get lost after n_i_ turns,

$$G_{i}=n_{i}G_{i}\left(1-\eta_{i}\right)\text{.}$$

From there, the number of turns of the size fraction i is,

$$n_{i}=1/\left(1-\eta_{i}\right)\text{.}$$

The cyclone efficiency η_i_ plays an important role to increase the number of turns n_i_, and hence, the residence time of the particles in a favourable combustion environment, and much work has been spent to improve this quantity.

**Fig. 3:**
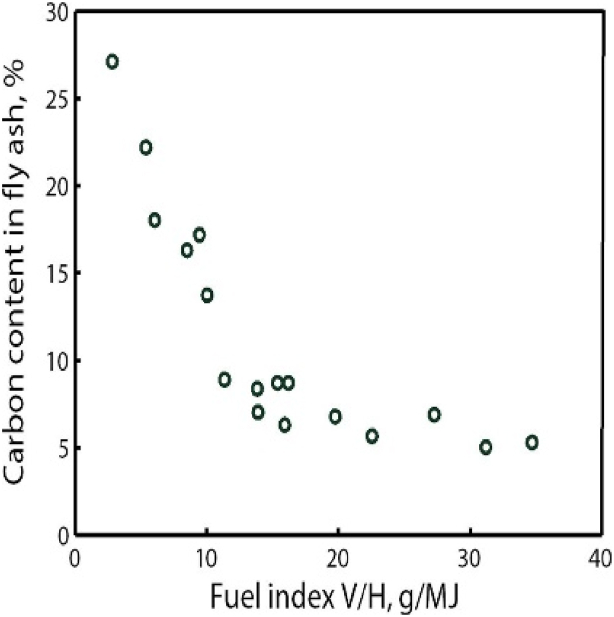
Carbon content in the fly ash versus a fuel index according to data for various Chinese coals, burnt in CFB boilers at an average bed temperature of 894°C and excess-air ratio of 1.25. The fuel index is the ratio of volatiles content (g/kg) based on dry and ash free fuel V and the lower heating value H MJ/kg, [9]. The data range from high rank coal (low fuel index) to low rank coal (high fuel index).

Furthermore, the combustion efficiency depends on the amount and reactivity of the char, as illustrated by Fig. 3 in the form of carbon content in the fly ashes (which is related to combustion efficiency) versus volatile content for several Chinese CFB boilers.

It can be assumed that the conditions in the boilers studied in Fig. 3 were relatively similar, so even if it is not possible to extract a combustion efficiency from the data, the figure illustrates the well-known fact that the reactivity of the fuels as well as their rank (here coals) increase with the content of volatiles.

**Fig. 4:**
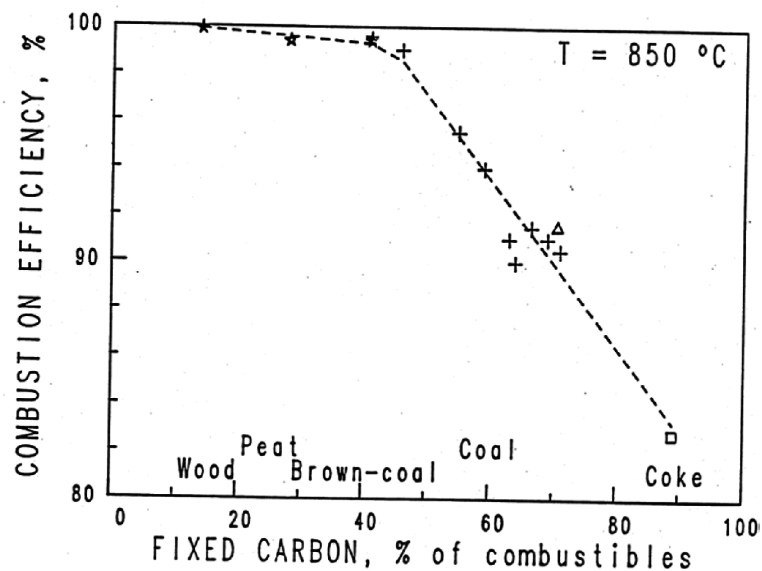
The influence of type of fuel on the combustion efficiency in BFB boilers [10].

The illustration shown in Fig. 4 on the significance of the volatile content (the combustible part = fixed carbon + volatile content) has been collected from BFB boilers. Obviously, the combustion efficiency is acceptable for high-volatile fuels like biomass, but unacceptably low for most coals.

**Fig. 5:**
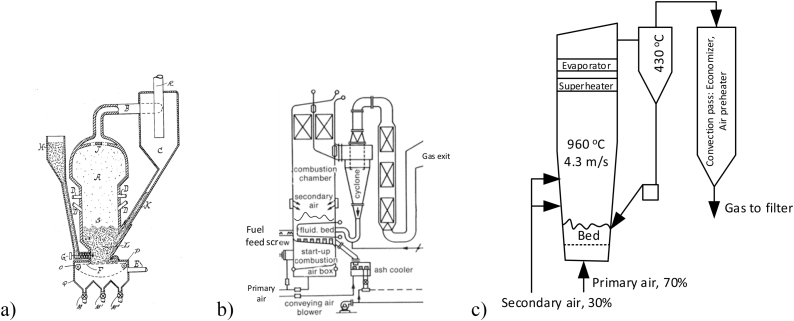
BFB converters with cyclones. a) From a patent by Winkler [11]; b) A German BFB boiler with ash recirculation from a cyclone [12]; c) The Circofluid boiler [13].

**Fig. 6:**
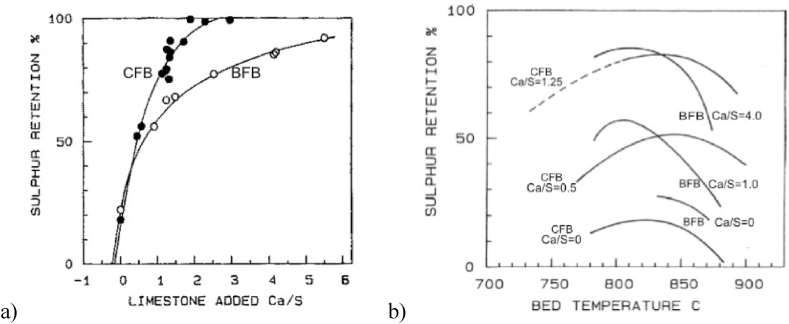
Comparison of sulphur capture with limestone in a BFB and a CFB boiler [14], a) Maximum sulphur retention as a function of limestone addition b) Temperature dependency of sulphur capture.

The dependency of combustion efficiency on fuel reactivity in a CFB is the same as in a BFB, but because of the prolonged residence time due to recirculation, the relationship seen in Fig. 4 will be displaced towards higher efficiencies in the case of CFB boilers.

The above can be summarized in several statements: 1) There is more unburned char in low-volatile fuels because of a larger quantity of fixed carbon in the fuel and usually a lower reactivity of the char. 2) in the BFB without recirculation of particles, the combustion efficiency of coal due to insufficient char combustion is unacceptably low. 3) With recirculation, the char residence time increases, and the char burnout improves for each turn of the recirculated particle flow. 4) The number of recirculation turns depend on the separator efficiency. 5) The residence time of the char also depends on the height of the transport zone in a combustor.

The difference between BFB and CFB with respect to the residence time and combustion efficiency is evident.

## 4 Particle circulation

Even from the beginning of the use of BFB it was realized that recirculation of elutriated bed material was an essential remedy for insufficient char conversion. Therefore, the developers of the BFB of Fig. 1a had installed a cyclone arrangement (small multi-cyclones) to recirculate the fly ash [6]. It soon became clear that the chosen cyclones were not suitable for high particle loading, not because of choking, but because of erosion on the unprotected cyclone walls. Consequently, recirculation could not be used with that particular arrangement.

Fig. 5 shows other early initiatives applying recirculation. The Winkler gasifiers had a very high loss of char in the fly ash, and already in 1928, Winkler submitted a patent application for a gasifier with a cyclone [11], Fig. 5a. However, most Winkler gasifiers up to 1975 were operated without recirculation, because the char-enriched ashes could be burned in nearby boilers [7]. The new development of Winkler gasifiers, called High-Temperature Winkler gasifier, introduced in 1976, did have a cyclone for ash and char recycling, thereby improving the efficiency of conversion. (The consequence of this improvement was that most ashes had to be removed as bottom ash. Since the char concentration is high in a bed of a gasifier operated with coal, the loss in combustibles due to the withdrawal of the ashes is still notable). The other BFB, shown in Fig. 5b–is also German. The ash handling system of this design went through several stages of development. In the final version, seen on the figure, the fly ash was captured by a cyclone and returned to the furnace. It was then a circulating system in a BFB boiler. The principal difference from the present CFB boilers is that the gas velocity, and hence the carry-over of particles, was less.

Fig. 5c shows a hybrid BFB-CFB of the name of Circofluid, developed by Deutsche Babcock in the end of the 1980s [13] and produced by licensees in the USA, India, and China, but it is not much noted on the market among other FB boilers. The fluidizing velocity is lower than that of a CFB but higher than that of a BFB. There are no cooling tubes in the bed, but the bed temperature is controlled by the air supply and recirculated ash. The furnace temperature is maintained at a sufficient level for combustion, controlled by the membrane-tube walls. Heat-transfer surfaces in the upper part of the furnace yield a gas temperature of 400–500 °C at the exit to the cyclone. This results in a smaller and lighter cyclone with less refractory than in the normal CFB boilers, which have a temperature of around 900 °C in the cyclone. Because of the low velocity, the particle concentration in the upper part of the furnace is lower than in a normal CFB, but, of course, the particles pass the tube bundles that must be well protected against erosion at the same time as they must fulfil their duty to exchange heat. There is little information available about the long-time performance of these tube bundles.

## 5 Emissions

The reason why there was a strong interest in FB combustion in the 1970s was the expected ability to reduce the most serious emissions at that time, those of sulphur (SO_2_) and nitrogen oxides (NO_x_, where x represents various oxides, notably NO and NO_2_).

### 5.1 Sulphur capture

Fig. 6 compares sulphur capture by limestone addition in the two BFB and CFB boilers of Fig. 1, operated with the same coal and the same limestone. Operation data are those shown in Table 1. Ca/S is the added calcium to sulphur molar ratio. There is also some calcium in the fuel, amounting to a molar ratio of 0.2–0.3, that explains the non-zero sulphur capture at the added quantity of Ca/S = 0. The results clearly illustrate that CFB is much more efficient in sulphur capture than BFB. Fig. 6a shows maximum values. The shape of the maxima is seen in Fig. 6b. This figure shows the well-known fact that sulphur capture in FB goes through a maximum at a bed temperature of about 800–850 °C. Also, in this respect a certain difference is noted between the two types of boilers: the maxima in the CFB occur at higher temperatures than in the BFB.

In an atmospheric FB, burning solid fuels, the added limestone calcines,

(1) CaCO_3_=CaO + CO_2_

and the resulting lime, CaO, reacts with SO_2_ under oxidising conditions:

(2) CaO + SO_2_ + ½O_2_=CaSO_4_.

Under reducing conditions CaO does not react with SO_2_, and the CaSO_4_ already formed may be reduced by CO, releasing sulphur:

(3) CaSO_4_ + CO=CaO + SO_2_ + CO_2_.

In extremely reducing cases CaS is formed instead of CaO.

There are at least two reasons for the differences in sulphur capture in the BFB and the CFB: 1) The recirculation of bed material in the CFB results in a longer residence time and better utilization of the limestone, 2) The bed of the BFB contains more reducing zones than the bottom bed of the CFB, and in the more oxidising freeboard of the BFB there are no particles to react with the SO_2_ that leaves the bottom bed in contrast to the situation in the CFB. CFB also has some tendencies for reducing zones in its bottom bed, but not so distinctly as in the BFB. Besides, in the CFB there is a considerable probability of reaction in the splash and transport zones of the furnace because of the simultaneous presence of oxygen and lime particles. The sulphur capture needs oxygen (Reaction 2) and lime particles. In contrast, if the gas and reacted lime, converted to CaSO_4_, meet a reducing environment, the reaction products may be CaS, or most likely the CaSO_4_ produced is converted back to CaO, and SO_2_ would be released (Reaction 3). This type of reactions may take place with a certain probability, depending on the degree of reducing or oxidising atmosphere that is present in the furnace, as discussed in Ref. [15]. Obviously, air staging, claimed above to be favourable for NO reduction, also has the negative influence of retarding combustion and sulphur capture in the bottom bed, owing to the tendency to increase the probability of reducing zones, both in a BFB and a CFB.

## 5.2 Nitrogen oxides

**Fig. 7:**
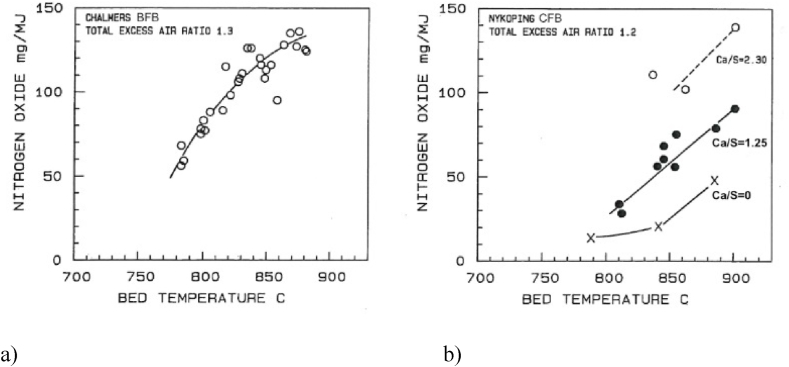
Comparison BFB-CFB. NO vs. bed temperature at different quantities of added limestone [14].

**Fig. 8:**
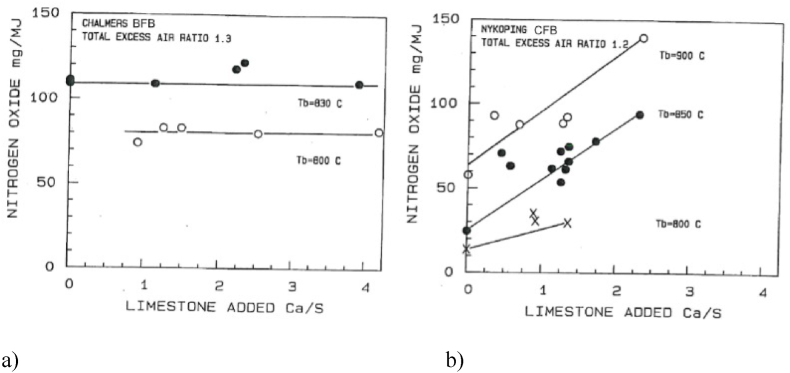
Comparison a) BFB b) CFB. NO vs. added limestone at different temperatures [14].

The comparison of measurements from the two boilers is shown versus bed temperature in Fig. 7 or versus added calcium to fuel-sulphur molar-ratio in Fig. 8. The emission of nitrogen oxide is measured as NO. There is almost no NO_2_ in an FB combustor, but the results are recalculated to NO_2_ for comparison with available emission standards. As could be expected, the emission increases with temperature almost with the same rate in both boilers. As not expected, there was an influence of limestone addition on the emission in the CFB but not in the BFB. The data points for added limestone to the CFB in Fig. 8b are represented by straight lines. A more careful representation shows a low value without limestone addition, and then a jump to a certain level as soon as limestone is added. The level is maintained about constant to Ca/S about two, where the sulphur capture was about complete as seen in Fig. 6a. At higher additions, the NO emission increases rapidly; the excess addition of limestone results in an exposure to unreacted lime, involving CaO. The influence of excess limestone addition was further confirmed by an NO emission of 232 mg/MJ at Ca/S = 3 (not shown in the diagrams).

A simplified analysis of the NO emission starts with the definition of emission as being the result of formation minus reduction during the residence time in the boiler. Likewise, one can conclude that predominantly,

(4) Char(N) → NO and N_2_

(5) Volatile(N) → NH_3_ and N_2_

Some formation and reduction reactions are presented in Table 2; two oxidation reactions (6 and 7) and one reduction reaction (8). For comparison, the table also shows the well-known reduction reaction (9) for ammonia injection, selective non-catalytic reduction (SNCR) of NO, active in in a certain temperature window without particles present.

**Table 2:** Reactions related to NO formation and destruction in an FB furnace [16].

Reaction	nr	BFB	CFB
NH_3_+5/4 O_2_→ NO + 3/2H_2_O	6	Oxidation on catalyst in the particle phase. Bypass of gas.	Oxidation on catalyst.
NH_3_+3/4 O_2_→ ½ N_2_ + 3/2H_2_O	7	Freeboard reaction.	Downstream of the cyclone: low particle concentration.
NO + CO → ½ N_2_+CO_2_	8	Reduction on catalyst.	Reduction on catalyst.
NH_3_ + NO + 1/4O_2_→ N_2_ + 3/2H_2_	9	SNCR, no particles.	SNCR, no particles.

All surfaces play some role as catalysts, either for oxidation or for destruction of ammonia. The most powerful ones are CaO and char.

Reaction 7 could lead to the production of N_2_, but it requires a higher temperature than those occurring in FB. CaO (but not significantly CaSO_4_) is acting as a catalyst in Reaction 6 to produce NO. Reduction of the NO by Reaction 8 could have char as a catalyst. The reaction is then more important in coal-fired FBs, having a high char loading (a few per cent of the bed), compared to biomass-fired beds where the char loading is very small. This is valid for both BFB and CFB.

**Fig. 9:**
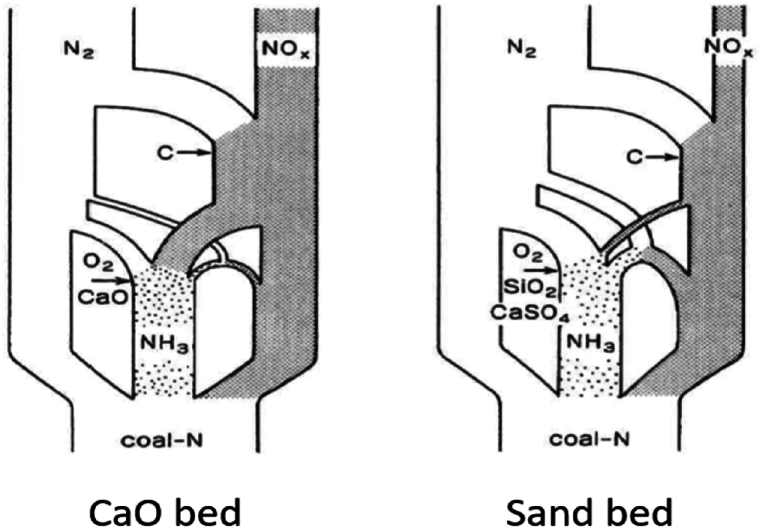
Flows of nitrogen during combustion of coal in an FB with a bed of CaO or of silica sand [17].

Hirama et al. [17] provided an explanation that helps to interpret the different NO emission behaviour in BFB and CFB, see Fig. 9.

The figure shows how the ammonia, released from devolatilization, may be affected by CaO to be converted to NO (Reaction 6) and only a minor fraction goes to N_2_. In a bed of sand or CaSO_4_, both predominantly non-catalytic, the ammonia is mostly converted to N_2_. The most important role of char in both beds is to reduce the NO formed to N_2_ (Reaction 8).

With the help of this information, the comparison of the nitrogen compounds observed in the BFB and the CFB can be interpreted tentatively. CaO plays a decisive role in CFB because of the simultaneous presence of CaO particles and oxygen in the expanded bottom bed and in the better mixed splash and transport zones. It remains to explain why the effect of lime is small in the BFB (Fig. 7a). The explanation is based on the same behaviour as discussed for sulphur capture. In the BFB, once the gases, including part of the ammonia, are released from the bed, reaching the freeboard, the possibility of meeting catalyst particles is limited. Also, there are more particles in the bubbles of a CFB than in a BFB, so reactions also could take place there in the CFB case, but less in the BFB case, enhanced by the fact that the particle phase in a BFB tends to be under reducing conditions. The particle phase of the bubbling bottom bed of a CFB shows similar features but is more ventilated than the BFB. In conclusion, the interaction of the ammonia gas with catalyst particles under oxidising conditions is less in a BFB than in a CFB. This result is clearly seen from the measurements.

## 6 Biomass and organic waste

In this section the ability of the BFB to burn high-volatile fuels like biofuel will be discussed. It is understood already from the beginning that these fuels can readily burn in a CFB, so the critical question is focussing on the ability of BFB to overcome the problems related to its design. Table 3 gives a summary.

**Table 3:** The solution of problems of operation with BFB.

BFB was found unsuitable for coal combustion, but it can be used for biomass and organic waste:
1)Erosion on in-bed heat-transfer tubes → volatiles burn in the freeboard, and this removes some of the heat release from the bed. Then the bed's heat balance can be closed at a reasonable bed temperature without tubes in the bed.
2)Unfavourable sulphur capture and low combustion efficiency → With biomass no sulphur capture is needed. The small fraction of char from biomass is quite reactive and yields a high combustion efficiency.
3)Unfavourable scale-up → The scale-up problem is avoided because transport to the plant is restricted in the case of biomass by its low energy density, J/m^3^ fuel.

### 6.1 The bed temperature

Of the heat supplied to the furnace (counted on 1 kg combustible fuel) a quantity is released in the bed, corresponding to a fraction of fuel φ. The energy content of the fuel is released in and above the bed,

$$H_{\text{LHV}}=\text{Heating}\;\text{of}\;\text{the}\;\text{gas}\;\text{in}\;\text{the}\;\text{bed}\;\varphi H_{\text{LHV}}+\text{heat}\;\text{released}\;\text{above}\;\text{the}\;\text{bed}\;\left(1-\varphi \right)H_{\text{LHV}}\text{.}$$

The heating of the gas flow g (kg/kg fuel) from the inlet temperature T_o_ the bed temperature T_bed_ with c_pm_ being the mean specific heat and without heat extraction by heat-transfer tubes,

$$\varphi H_{\text{LHV}}={\text{gc}}_{\text{pm}}\left(T_{\text{bed}}-T_{o}\right)$$

This gives the bed temperature,

$$T_{\text{bed}}=\varphi H_{L\text{HV}}/\left({\text{gc}}_{\text{pm}}\right)\;–\;T_{o}\text{.}$$

φ = 1 yields a bed temperature T_bed_ equal to the adiabatic temperature. Because of over-bed combustion of volatiles, the factor φ is < 1 and such that the bed temperature is reasonable without cooling tubes immersed in the bed. Hence, the problem of tube erosion is avoided. Biofuels and many waste fuels have a volatile content of 80 %, leaving 20 % for char. The over-bed combustion takes place because of the tendency of the volatiles to leave the bed without being burnt. This can be further controled by the supply of primary air, creating a certain stoichiometry in the bed, lower than that of complete combustion. The biomass char is more reactive than coal char in general, which also facilitates the mode of operation suggested. The corresponding combustion efficiency of a BFB boiler (bed and freeboard) can be read from Fig. 4. It is almost 100 % without recirculation. With part of the heat released in the freeboard, relying mostly on its walls for heat transfer, secondary air is supplied with care to avoid hot spots. In such cases the secondary-air supply could be split into air-supply nozzles at several altitudes in the furnace. Obviously, for this type of fuel BFB operates well.

### 6.2 The scale-up problem

Biomass is not suitable for transportation. Therefore, usually the biomass plant is small, utilizing only fuel from the neighbourhood of the plant (<100 km), and the scale-up problem is avoided. The data in Table 4, expressing the volumes of various forms of biomass compared to the volume of coal for the same energy content, illustrate this aspect.

**Table 4:** Bulk volumes of biomass fuels compared to coal.

Fuel	Ash %	Moisture %	Volume (m^3^/m^3^ coal)_MJ_
Coal	10	10	1
Wood pellets	1	5	2
Wood powder	1	5	4
Wood chips	1	50	7
Saw dust	1	50	9
Bark	1	50	8
Straw briquettes	5	<18	3
Straw bale	5	<18	13
Straw natural	5	<18	20

For the same quantity of energy, the volume of biomass is several times greater than coal, and even the compressed forms, such as pellets and briquettes, have considerable volumes. This is not suitable for long-range transport, even in these most favourable cases. Furthermore, an elaborated form of fuel like pellets or briquettes is more expensive than the simpler forms, like chips. The conclusion is that biomass and waste are local in the first place, and only in extreme situations they are transported longer distances. Consequently, most of the related boilers are small, below a few hundred MW_th_.

## 7 A comment on high-volatile fuels

The control of the biomass bed by moderating the air supply, mentioned above, was done “spontaneously” as a reasonable action to maintain the desired bed temperature. However, this action was introduced already in the first gasifiers for the same reason: to maintain a chosen bed temperature. Other high-volatile fuels like oil shale behave in a similar way. The very first BFB combustor for the conversion of Estonian oil shale was a laboratory unit consisting of a bottom part for primary conversion (devolatilization) of the solid fuel and an upper part for combustion of the gases and some elutriated char with secondary air, successfully tested in 1951 and followed by larger-scale combustors [18]. The combustible matter of oil shale consists of more than 80 % volatiles. Its ash content is high. The combustion in the primary part of the reactor aimed at attaining a suitable temperature for heating and devolatilizing of the fuel. In the secondary part of the reactor, where the gases were burned by added secondary air, there were no heat transfer devices in this first test reactor and the temperature tended to be high or the reactor had to be cooled by excess air. In later equipment, heat-transfer surfaces were installed. A gradual improvement with further investigations is mentioned, but in the present review the purpose is only to point out the similarity between this first combustor for high-volatile fuel and the biomass BFB mentioned above.

## 8 Multi-reactor configurations

Combinations of reactors, CFB-BFB or CFB-CFB are sometimes made to achieve certain goals. In this presentation the focus is on conversion of solid fuels (mostly combustion in boilers). Arrangements of FB reactors for other purposes, for instance mentioned by Kunii and Levenspiel [19], are not included here, even if such devices may have served as a source of inspiration for designs. This also excludes reactors based on fluidized beds for flue-gas cleaning like calcium looping or adsorption-desorption systems, although, admittedly, such devices are of interest in many developments using fluidized bed beside the principal focus here, which is boilers.

Almost unexpected, the common CFB boiler with an external heat exchanger (EHEX) belongs to this group. An EHEX is a BFB with inserted tube bundles to extract heat. In this application erosion is not as severe as in a BFB boiler for two reasons: 1) the circulating particles flowing through the EHEX are small (100–200 μm [20]), 2) the fluidization velocity is low, only a few times u_mf_.

Initially, there were many proposals for CFB boilers, for instance by Ref. [21], consisting of a refractory-lined reactor for combustion, proposed to operate in the fast-fluidization regime. The entire cooling was suggested to take place in an external FB, a “slow” bed (a BFB), where heat-transfer surfaces were installed. There were several similar proposals at the time, but the patent of Lurgi [22], including the combination of a CFB riser-combustor and an EHEX in a BFB mode, became the basis for the dominant CFB design in the following years.

**Fig. 10:**
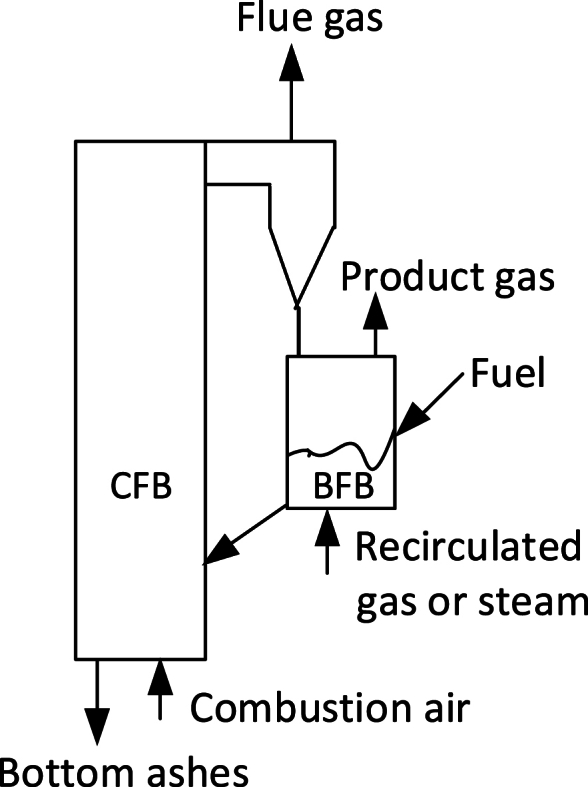
A combined CFB-BFB system.

The BFB-CFB combination also proved useful for several fuel conversion tasks, such as indirect gasification of high-volatile fuels like biomass. Fig. 10 shows a simplified sketch of such a plant, implemented, for instance at Güssing, Austria [23], to produce heat to a district-heating system and gas for electric power, generated by gas engines. The fuel is fed to the bubbling bed that serves as a devolatilizer, receiving its heat from the combustion of the residual char in the CFB riser. In this way the nitrogen in the combustion air escapes as flue gas from the CFB and does not dilute the product gas leaving the BFB.

A similar system could solve the shale-combustion problem mentioned above, where there was no clear separation between the combustion of devolatilized gas and residual char. The shale would be fed to the bubbling bed, such as in Fig. 10, for the release of gas, whereas the char (called semicoke in this context) could be transported from the bubbling bed to the riser for combustion. In this case “the wheel was already invented”: starting in the 1940s with a proposal to use the semicoke and ashes, burned in an entrained-flow reactor to provide heat for devolatilization of the fresh oil shale in a “retort”. Following the tradition in the oil-shale utilization industry, the retort was (and remains so in present plants) a rotary kiln. Disregarding various possible minor problems of development, this retort could have been a bubbling bed as indicated in Fig. 10, and the riser reactor could have been a CFB. Indeed, in the recent development the riser reactor has been designed as an adiabatic CFB reactor, but the retort is still a rotary kiln [24]. The rotary kiln-CFB plant, operating in Estonia, treats 280 tonnes/h of shale and is installed under the trade name of Enefit-280 in a power plant in Narva.

Actually, for this particular purpose (degasification) the rotary kiln has the advantage of being a pseudo-plug-flow reactor, leaving degasified ashes and semicoke at the exit, while a BFB, and even to some extent a CFB, is a pseudo well-mixed reactor whose exit flow of solids contains a mixture of fully and partly degasified fuel.

Another interesting example of a combination of a CFB-BFB (which could also be CFB-CFB) is in chemical looping combustion. In this case the CFB reactor is called “Air reactor” and serves to oxidize the bed material that consists of metal particles. The bed material is circulated to the other reactor, which can be a bubbling or circulating reactor, called “Fuel reactor” where the fuel particles are introduced to be converted by the oxygen brought there by the metal particles. A survey of the development of this device is given in Ref. [25].

It is obvious from Fig. 10 and the above descriptions that in a twin or multiple reactor system, the CFB has an additional function in transporting the bed material from one reactor to another.

## 9 Conclusions

• CFB and BFB boilers are just two extremes of the same FB principle. Circulation of bed material is the most outstanding difference between the two concepts.
• Cooling tubes in contact with a bubbling combustion bed are unsuitable, and the insufficient cooling of the bed excludes coal combustion in a BFB.
• Without cooling tubes in the bed, such as during combustion of high-volatile fuels, BFB can be used as well as CFB. The cost and size decide which type of boiler to choose.
• Performance features favour CFB in comparison to BFB for all fuels. For CFB:
  - ----heat-transfer surfaces are in low-density areas (dominated by radiation) with less erosion;
  - ----recirculation of char or lime improves the efficiency of sulphur capture and combustion.

With biomass and high-volatile waste the above two advantages of coal utilization are less important.

• Combination of BFB and CFB has many advantages. A few examples within the framework of solid fuel conversion have been mentioned: external heat-exchangers for CFB, chemical looping combustion, conversion of oil shale, and indirect gasification of biomass.

## Funding

This work did not receive any specific grant from funding agencies in the public, commercial, or non-for-profit sectors.

## Data availability

No dara were used for the research described in this article.

## CRediT authorship contribution statement

**Bo Leckner:** Writing – review & editing, Writing – original draft, Visualization, Validation, Software, Resources, Project administration, Methodology, Investigation, Formal analysis, Data curation, Conceptualization.

## Declaration of competing interest

The author declares that are no known competing financial interests or personal relationships that could have appeared to influence the work reported in this paper.
